# Decreasing incidence of pregnancy among HIV-positive adolescents in a large HIV treatment program in western Kenya between 2005 and 2017: a retrospective cohort study

**DOI:** 10.1186/s12978-020-01031-5

**Published:** 2020-12-02

**Authors:** Heather C. Millar, Alfred K. Keter, Beverly S. Musick, Edith Apondi, Juddy Wachira, Katherine R. MacDonald, Rachel F. Spitzer, Paula Braitstein

**Affiliations:** 1 Academic Model Providing Access to Healthcare (AMPATH), PO Box 4606, Eldoret, 30100 Kenya; 2 Section of Gynaecology, Division of Endocrinology, SickKids Hospital, 555 University Avenue, 7th Floor, Black Wing, Toronto, ON M5G 1X8 Canada; 3 Department of Obstetrics and Gynaecology, University of Toronto Faculty of Medicine, 123 Edward Street, Suite 1200, Toronto, ON M5G 1E2 Canada; 4 Department of Biostatistics, Indiana University School of Medicine, 410 West 10th Street, Suite 3000, Indianapolis, IN 46202 USA; 5 Moi Teaching and Referral Hospital, Nandi Road, Uasin Gishu County, PO Box 3-30100, Eldoret, Kenya; 6 Department of Behavioral Sciences, Moi University, College of Health Sciences, PO Box 4606, Eldoret, 30100 Kenya; 7 Department of Pediatrics, Indiana University School of Medicine, 705 Riley Hospital Drive, Riley Hospital 5900, Indianapolis, IN 46202 USA; 8 Department of Epidemiology, University of Toronto Dalla Lana School of Public Health, Health Sciences Building, 155 College Street, 6th Floor, Toronto, ON M5T 3M7 Canada; 9 Moi University, College of Health Sciences, School of Medicine, PO Box 4606, Eldoret, 30100 Kenya

**Keywords:** Pregnancy in adolescence, Adolescent, HIV, Pregnancy, Contraception, Family planning services

## Abstract

**Background:**

The objective of this study was to estimate the prevalence, incidence and risk factors for pregnancy among HIV-positive adolescents in a large HIV treatment program in western Kenya.

**Methods:**

The Academic Model Providing Access to Healthcare (AMPATH) program is a partnership between Moi University, Moi Teaching and Referral Hospital and a consortium of 11 North American academic institutions. AMPATH currently provides care to 85,000 HIV-positive individuals in western Kenya. Included in this analysis were adolescents aged 10–19 enrolled in AMPATH between January 2005 and February 2017. Socio-demographic, behavioural, and clinical data at baseline and time-updated antiretroviral treatment (ART) data were extracted from the electronic medical records and summarized using descriptive statistics. Follow up time was defined as time of inclusion in the cohort until the date of first pregnancy or age 20, loss to follow up, death, or administrative censoring. Adolescent pregnancy rates and associated risk factors were determined.

**Results:**

There were 8565 adolescents eligible for analysis. Median age at enrolment in HIV care was 14.0 years. Only 17.7% had electricity at home and 14.4% had piped water, both indicators of a high level of poverty. 12.9% (1104) were pregnant at study inclusion. Of those not pregnant at enrolment, 5.6% (448) became pregnant at least once during follow-up. Another 1.0% (78) were pregnant at inclusion and became pregnant again during follow-up. The overall pregnancy incidence rate was 21.9 per 1000 woman years or 55.8 pregnancies per 1000 women. Between 2005 and 2017, pregnancy rates have decreased. Adolescents who became pregnant in follow-up were more likely to be older, to be married or living with a partner and to have at least one child already and less likely to be using family planning.

**Conclusions:**

A considerable number of these HIV-positive adolescents presented at enrolment into HIV care as pregnant and many became pregnant as adolescents during follow-up. Pregnancy rates remain high but have decreased from 2005 to 2017. Adolescent-focused sexual and reproductive health and ante/postnatal care programs may have the potential to improve maternal and neonatal outcomes as well as further decrease pregnancy rates in this high-risk group.

## Plain English summary

In this study we looked at how many HIV positive adolescent girls in a large HIV treatment program in western Kenya became pregnant from 2005 to 2017. There were 8565 adolescent girls who received care in our program during that time. The average age that they signed up for the care program was 14 years. Only 17.7% of these girls had electricity at home and 14.4% had piped water, which told us that many of these girls were poor. 12.9% of these girls were pregnant when they joined the care program. Of those who were not pregnant when they started in our HIV care program, 5.6% became pregnant later. 1.0% of the girls were pregnant when they joined the care program and then became pregnant again another time while they were still under the age of 20 years. There were 55.8 pregnancies for every 1000 adolescents in the study. Adolescents who got pregnant were more likely to be older, to be married or living with a partner and to have at least one child already and less likely to be using birth control. Between 2005 and 2017, the number of adolescents who got pregnant did decrease overall but was still much higher than adolescents in wealthy countries.

Our conclusions from this study are that many adolescents with HIV in western Kenya are becoming pregnant and that more attention should be given to adolescent-focused sexual health and pregnancy care programs.

## Background

There are an estimated 21 million pregnancies per year among adolescents in low and middle income countries, with 50% of these being unintended [1]. Since the 1990s, the adolescent birth rate has been decreasing but, in Sub-Saharan Africa, this decrease has been slow and, overall, the adolescent birth rate remains high, with a change from 139 births per 1000 girls aged 15–19 between 1990 and 1995 to 109 births per 1000 girls aged 15–19 between 2010 and 2015 [2]. In Kenya, where 47% of women have begun child-bearing by age 20, recent estimates suggest an increase in adolescent pregnancy rates from 96 births per 1000 women aged 15–19 in 2008 to 121 births per 1000 women aged 15–19 in 2014 [3, 4]. This is despite the release of a national Adolescent Reproductive Health and Development Policy in 2003, which called for the development of adolescent-focused health services and specifically targeted a reduction in the proportion of women below age 20 with a first birth from 45% in 1998 to 22% by 2015 [5]. With this lack of progress has come a renewed call for evidence-based provision of adolescent-focused sexual and reproductive health services and pregnancy prevention, with HIV positive adolescents identified as a specific vulnerable population [3, 6, 7].

Pregnancy in adolescence is associated with increased maternal and neonatal mortality and morbidity, including increased rates of obstetric fistula, anemia, unsafe abortion, preterm birth and low birth weight [6, 8–12]. In addition, adolescent mothers are at risk for depression and stress associated with pregnancy [12–15]. In the longer term, these young women are less likely to complete their education, obtain sustainable employment and, as a consequence, are less likely to be able to lift themselves and their families out of poverty [6]. Adolescent pregnancy itself can be a source of increasing inequality within communities [11]. It therefore has important negative consequences, not only for the individual woman and her child, but also, for the future health and well-being of communities and countries [6].

Pregnancy in adolescence is itself an indication of a lack of access to equal rights and opportunities for girls and young women and a lack of control over their sexual and reproductive health. Nine out of ten adolescent pregnancies globally occur in the setting of marriage, indicating ongoing high rates of child marriage worldwide. Pregnancy is more likely in girls who are poor and no longer in school [6]. Adolescent mothers routinely describe their pregnancy as unplanned, indicating a lack of access to contraception and lack of control over sexual decision-making [16]. Pregnancy may be the result of intimate partner violence, which is experienced by approximately 30% of girls aged 15–19 worldwide [17].

HIV positive adolescents who are pregnant represent a high-risk group who may have little control over their own sexual and reproductive health and who are at risk for additional health and social consequences. In a study of HIV positive adolescents in Nairobi and Nyanza provinces in Kenya, 44% of girls described their first sexual experience as being associated with rape/forced, being tricked or persuasion with money [18]. Males were less likely than females to disclose their HIV status to their partners and were more likely to have HIV-negative partners. Two-thirds of adolescent girls who had been sexually active had been pregnant and 75% of these pregnancies were unintended [18]. These high rates of unintended pregnancy provide evidence of inadequate access to appropriate contraception and prevention of STI transmission, including HIV. HIV positive pregnant adolescents have been shown to be more likely than adult women to only become aware of their HIV diagnosis at presentation to antenatal care [8]. In addition, rates of maternal to child transmission of HIV are increased in HIV positive adolescents in comparison to adults due to lower uptake of Prevention of Mother to Child Transmission (PMTCT) [8, 19, 20]. Adolescents are therefore a critical target group in preventing propagation of the HIV epidemic through vertical transmission. Finally, HIV positive pregnant adolescents have an increased need for social support during pregnancy, due to the stigma of both adolescent pregnancy and HIV as well as the requirement for ART compliance individually and for a newborn child [3, 20]. These adolescents are often already vulnerable, due to poverty, homelessness and orphan status [18, 21].

The aim of this study was to estimate the prevalence, incidence and risk factors for pregnancy among HIV-positive adolescent girls in a large HIV treatment and care program in western Kenya, in order to improve Adolescent Sexual and Reproductive Health-targeted services within the program and for its catchment population. By describing trends in pregnancy incidence over time as well as specific high risk factors, care providers and program managers can then use this data to advocate for increased resources for adolescent pregnancy prevention and care and target these resources to those most at risk.

## Methods

### Setting

The Academic Model Providing Access to Healthcare (AMPATH) program is a partnership between Moi University and Moi Teaching and Referral Hospital in Eldoret, Kenya and a consortium of 11 North American Academic Institutions. AMPATH works within the public health care system in Kenya and currently provides care to 85,000 HIV-positive individuals in the western part of the country. This study took place in the following counties: Bungoma, Busia, Elgeyo-Marakwet, Kisumu, Nandi, Trans-Nzoia, Uasin Gishu, and West Pokot. AMPATH is involved in inpatient and outpatient clinical care, while also working on a range of population and community health initiatives. To date, the program has not formally assessed the burden and needs of its HIV-positive female adolescent population with regards to sexual health and pregnancy.

### Study design, participants and data collection

This study was a retrospective cohort study of HIV-positive adolescent girls aged 10–19 enrolled in AMPATH between January 1, 2005 and February 28, 2017. Socio-demographic, behavioural, clinical and time-updated antiretroviral treatment (ART) data were extracted from the electronic medical records and summarized using descriptive statistics. In the AMPATH program, clinical data is collected on paper forms at each patient encounter by health providers. These data are then entered into an electronic database by data entry clerks. There are separate patient encounter forms for paediatric, adult and antenatal clinics and some concepts such as marital status and family planning are not included on the paediatric forms.

### Data analysis

Baseline characteristics were defined as the participant characteristics at the time of inclusion in the study cohort. For those whose enrolment in AMPATH care occurred between the ages of 10 and 19, baseline characteristics reflect characteristics at enrolment. For those who enrolled in AMPATH care prior to 10 years of age, baseline characteristics reflect characteristics at age 10, when the participant became part of the cohort. Clinical characteristics at the time of conception included CD4 cell count per mm^3^, WHO clinical stage, body mass index (BMI) (kg/m^2^), and hemoglobin (g/dL). With the exception of CD4, these variables were defined with a window of 90 days prior to and 30 days post estimated conception date among those who became pregnant or 90 days prior to the last visit date among those who did not become pregnant. CD4 was defined with a window of 180 days prior to and 30 days post estimated conception date among those who became pregnant or 180 days prior to the last visit date among those who did not become pregnant. Approximate date of conception was determined using pregnancy-related measures such as date of last menstrual period (LMP), estimated or actual date of delivery, pregnancy status at each clinic visit and gestational age. Follow up time was defined as time of inclusion in the cohort until the date of first pregnancy or age 20, loss to follow up, death, or patient’s last visit. Participants who were pregnant and remained in care after delivery were re-initiated into the study at date of delivery. While in reality a woman cannot conceive immediately following delivery, due to sometimes inaccurate delivery dates in the medical record, this strategy was chosen as the most consistent approach to re-initiation.

Data analysis was done using SAS 9.4. Categorical variables such as WHO clinical stage, clinic location, disclosure status, hospitalization status, orphan status, and pregnancy status among others were summarized using frequencies and the corresponding percentages. Continuous variables were summarized using median and the corresponding inter quartile range (IQR) when they were found to violate the Gaussian assumptions. The Gaussian assumptions were assessed using Shapiro–Wilk test and histograms.

Association between pregnancy status and categorical variables was assessed using Pearson’s Chi Square test. The association between pregnancy status and continuous variables was assessed using two sample Wilcoxon-ranks sum test. Kaplan–Meier survival function was used to describe the rate of pregnancy over time. Cox proportional hazards regression model was used to assess the factors associated with pregnancy. We reported the hazard ratios and the corresponding 95% confidence intervals (95% CI).

For descriptive analysis, complete cases analysis was performed for every variable (i.e. we described the participants who had observed data for each specific variable). For the regression models, missing data was treated as a separate category. For example, if for WHO clinical stage some participants had missing data then there were five categories: WHO clinical stages I, II, III, IV and a “Missing Data” group. This was done to avoid losing cases of pregnancies and, thus, enhance the power of the study.

### Ethics

Ethics approval was obtained through the Moi University School of Medicine Institutional Research Ethics Committee and the University of Toronto Health Sciences Research Ethics Board, as part of an overall approval for retrospective analysis of the AMPATH Medical Records System (AMRS) aimed at improving clinical care. Included in this ethics approval is a waiver of informed consent, due to the retrospective nature of the analysis.

## Results

There are 8565 female participants 10–19 years old who visited an AMPATH clinic between January 1, 2005 and February 28, 2017. Table 1 shows the demographic and socio-economic characteristics of these 8565 adolescents. The median age at enrolment in the AMPATH HIV care program was 14.0 years (IQR 10.0, 18.4). Slightly more than half (56.8%, 4865/8565) of these adolescent girls were receiving care in an urban clinic, with 43.2% (3700/8565) in rural clinics and 25.3% (682/2698) of participants having to travel more than one hour to reach the clinic. A small percentage had electricity (17.7%, 700/3949) or piped water (14.4%, 503/3495) at home, and approximately half (49.5%, 1982/4001) were orphans. A significant proportion (35.1%, 1571/4476) of these adolescent girls were married or living with a partner, with 23.5% (481/2046) suspecting infidelity by their spouse. Half (50.6%, 1259/2486) of these girls had been sexually active within 6 months of enrolment into HIV care. At baseline, 53.5% (2326/4345) of the sample had disclosed their HIV status and, at some point during their follow-up, 82.3% (7051/8565) were initiated on Anti-Retroviral Therapy (ART). When asked about family planning use, only 2.7% (171/6409) described not being sexually active as a qualifier for not using. 40.8% (2618/6409) had ever used family planning. However, out of 4664 for whom data was available on type of family planning method, 83.1% were not using any method.

**Table 1:** Socio-demographic and clinical characteristics at enrollment (N = 8565)

Variable	N	Median (IQR) or n (%)
Age at study inclusion (years), Median (IQR)	8565	14.0 (10.0, 18.4)
Urban clinic location, n (%)	8565	4865 (56.8)
Have electricity at home^a^	3949	700 (17.7)
Have piped water at home^a^	3495	503 (14.4)
Orphan (mother or both parents dead)	4001	1982 (49.5)
Married or living with a partner, n (%)	4476	1571 (35.1)
Had sex outside marriage^b^	2103	178 (8.5)
Suspects spouse to be having sex outside of marriage^b^	2046	481 (23.5)
Had sex within 6 months of enrollment into HIV care^b^	2486	1259 (50.6)
Travel time to clinic (min)^b^	2698	
< 30		1054 (39.1)
30–60		962 (35.7)
60–120		447 (16.6)
> 120		235 (8.7)
Have disclosed HIV status		
Overall	4345	2326 (53.5)
Hospitalized in the year prior to inclusion	7114	630 (8.9)
Ever initiated ART	8565	7051 (82.3)
Ever used family planning^c^	6409	
No, Not sexually active		171 (2.7)
No		3620 (56.4)
Yes		2618 (40.8)
Age when first family planning data recorded	6409	17.2 (14.4, 19.0)
Family planning method^d^	4664	
None		3875 (83.1)
Condoms		513 (11.0)
Female condoms		4 (0.1)
Emergency contraceptive		1 (0.02)
Implant contraceptive		83 (1.8)
Oral contraceptive		17 (0.4)
IUD		20 (0.4)
Natural family planning		3 (0.1)
Injectable contraceptive		155 (3.3)
Other method		13 (0.3)

^a^Adult encounters only; collection discontinued in 2015

^b^Adult encounters only; collection discontinued in 2013

^c^Family planning use includes use of female sterilization, contraceptive implant, intrauterine device, injectable contraception, oral contraceptive pills, emergency contraception, condoms, diaphragms, or natural family planning methods

^d^Close to conception data (6 months pre- and 1 month post- for those who became pregnant, and 6 months before last visit for those who were not pregnant). Adolescents may contribute to more than one category

Of the 8565 adolescent girls in the study, 12.9% (1104/8565) (or 129 per 1000 women) were pregnant at the time of inclusion in the study cohort (Table 2). During follow-up, 5.6% (448/8565) (or 56 per 1000 women) became pregnant, while still less than 20 years of age. Furthermore, 1.0% (78/8565) were pregnant at the time of inclusion and became pregnant again during follow-up. The median age at conception among those who became pregnant during follow up was 18.7 years (IQR 17.5, 19.4).

**Table 2:** Pregnancy characteristics

Variable	n	Median (IQR) or n (%)
Pregnant at the time of inclusion in the study	8565	1104 (12.9)
Age at conception among those pregnant at study inclusion (years)	1104	18.8 (17.8, 19.5)
Pregnant during follow up	8565	448 (5.6)
Pregnant at the time of inclusion in the study and again during follow up	8565	78 (1.0)
Age at conception among those pregnant during follow up (years)	448	18.7 (17.5, 19.4)

The new pregnancies during follow-up in care result in an overall incidence rate of 21.9 (95% CI 20.0, 24.0) pregnancies per 1000 woman years, with 20,466.4 person years of follow up (Table 3). Excluding the 541 adolescents who were pregnant at inclusion and did not continue in care post-partum, the median follow-up time was 1.52 years (IQR: 0.37 to 4.15 years), with the shortest follow-up time as 0 years and the longest 10 years. The incidence rates by age range from 1.3 to 87.5 pregnancies per 1000 woman years between the ages of < 15 to 19. The highest incidence was at age 18, with an incidence rate of 87.5 per 1000 woman years. There were 15 new pregnancies among girls < 15 years of age. When analyzed by calendar year, incidence rates overall show a decrease over time, with a rate of 52.6 per 1000 woman years in 2005 to 8.9 per 1000 woman years in 2017 (Table 4). Pregnancies per 1000 women also decreased over time, from 23.2 per 1000 women in 2005 to 8.4 per 1000 women in 2017.

**Table 3:** Incidence rates of pregnancy by age at conception

	Total at risk	Number of new pregnancies	Pregnancies per 1000 women	Number of person years of follow up	Incidence rate per 1000 woman years (95% CI)
Overall	8024^a^	448	55.8	20,466.4	21.9 (20.0, 24.0)
Age at conception					
< 15	4563	15	3.3	11,740.8	1.3 (0.8, 2.2)
15	2202	28	12.7	1901.6	14.7 (10.1, 21.3)
16	1984	42	21.2	1608.8	26.1 (19.3, 35.3)
17	2017	66	32.7	1502.9	43.9 (34.5, 55.9)
18	2220	127	57.2	1450.7	87.5 (73.5, 104.1)
19	2824	170	60.2	2261.6	64.7(64.7, 87.4)

^a^541 adolescents who were pregnant at study entry did not contribute any time at risk as they did not have any follow up within the care program after delivery

**Table 4:** Incidence rates of pregnancy by year

Year of pregnancy start	Total at risk	Number of incident pregnancies	Pregnancies per 1000 women	Number of person years of follow up	Incidence rate per 1000 woman years (95% CI)
Overall	8024	448	55.8	20,466.4	21.9 (20.0, 24.0)
2005	301	7	23.3	133.1	52.6 (25.1, 110.3)
2006	612	23	37.6	309.9	74.2 (49.3, 111.7)
2007	926	34	36.7	554.9	61.3 (43.8, 85.8)
2008	1100	25	22.7	758.4	33.0 (22.3, 48.8)
2009	1398	30	21.5	1022.8	29.4 (20.6, 42.0)
2010	1698	39	23.0	1270.5	30.7 (22.4, 42.0)
2011	2106	60	28.5	1663.59	36.1 (28.0, 46.5)
2012	2428	47	19.4	1973.7	23.8 (17.9, 31.7)
2013	2649	37	14.0	2055.2	18.0 (13.0, 24.8)
2014	2634	59	22.4	2306.3	25.6 (19.8, 33.0)
2015	2767	35	12.6	2494.7	14.0 (10.1, 19.5)
2016	2938	27	9.2	2761.5	9.8 (6.7, 14.3)
2017^a^	2985	25	8.4	2818.5	8.9 (6.0, 13.2)

^a^Includes only until Feb 28, 2017

Finally, Table 5 shows factors associated with becoming pregnant during follow-up. Adolescents who became pregnant in follow-up were more likely to be older (AHR: 1.34, 95% CI 1.26, 1.43, per year increase), to be married or living with a partner (AHR: 1.35, 95% CI 1.05, 1.73), to have at least one child at enrolment (AHR: 2.01, 95% CI 1.54, 2.62) and to have disclosed their HIV status (AHR: 1.33, 95% CI 1.02. 1.73). A decreased risk of pregnancy was found in those with WHO clinical stage of 3 in comparison to 1 (AHR: 0.71, 95% CI 0.52, 0.98), WHO clinical stage 4 in comparison to 1 (AHR: 0.33, 95% CI 0.13, 0.81), and family planning use (AHR: 0.75, 95% CI 0.57, 0.97).

**Table 5:** Unadjusted and adjusted hazard ratios (HR) and 95% confidence intervals (CI) of incident pregnancy among the 8024 adolescents

Variable	Unadjusted HR (95% CI)	Adjusted HR (95% CI)
Age at enrollment (years)	1.75 (1.66, 1.84)	1.34 (1.26, 1.43)
Married or living with a partner		
Yes vs. no	3.19 (2.56, 3.99)	1.35 (1.05, 1.73)
Had sex within 6 months prior to enrollment		
Yes vs. no	2.28 (1.77, 2.93)	1.26 (0.96, 1.64)
Had sex outside of marriage		
Yes vs. no	1.93 (1.28, 2.91)	1.36 (0.90, 2.07)
Urban clinic location		
Yes vs. no	0.90 (0.75, 1.08)	0.97 (0.80, 1.17)
Pregnant at the time of inclusion in the study		
Yes vs. no	6.05 (4.68, 7.82)	1.12 (0.85, 1.47)
Had at least one child at enrollment		
Yes vs. no	4.87 (3.79, 6.25)	2.01 (1.54, 2.62)
HIV status disclosed at enrollment		
Yes vs. no	4.94 (3.86, 6.31)	1.33 (1.02, 1.73)
Hospitalized in the year prior to enrollment		
Yes vs. no	0.50 (0.33, 0.77)	0.71 (0.46, 1.09)
^a^CD4 cell count per mm^3^		
> 350 vs. ≤ 350	1.56 (1.15, 2.12)	1.26 (0.91, 1.74)
^b^ BMI (kg/m^2^)		
18.5–25 vs. ≤ 18.5	4.94 (2.99, 8.19)	1.88 (1.12, 3.13)
> 25 vs. < 18.5	2.56 (1.01, 6.45)	0.72 (0.28, 1.84)
^b^ Hemoglobin (g/dL)		
> 11 vs. ≤ 11.0	2.14 (1.13, 4.08)	1.54 (0.80, 2.95)
^b^WHO clinical stage		
2 vs. 1	0.61 (0.45, 0.81)	0.87 (0.64, 1.18)
3 vs. 1	0.40 (0.30, 0.54)	0.71 (0.52, 0.98)
4 vs. 1	0.18 (0.08, 0.45)	0.33 (0.13, 0.81)
Family planning use		
Yes vs. no	2.48 (1.93, 3.18)	0.75 (0.57, 0.97)

*HR* hazard ratio, *CL* confidence limits

^a^Close to conception data (6 months pre- and 1 month post- for those who became pregnant, and 6 months before last visit for those who were not pregnant)

^b^Close to conception data (3 months pre- and 1 month post- for those who became pregnant, and 3 months before last visit for those who were not pregnant)

## Discussion

Consistent with much of the literature on adolescent pregnancy, the adolescents in this cohort were of low-income status, with low rates of electricity and piped water in their homes as surrogate measures of poverty. Further vulnerability is indicated by the high percentage who were orphans. The sample had almost equal representation from both urban and rural areas, but many travelled long distances to reach care. One third of the participants were married or living with a partner and a quarter felt that their partners were having sex outside of marriage, indicating possible unstable and unsafe relationships, with risk of further HIV transmission. Half of participants had been sexually active within 6 months of enrolment in HIV care, highlighting the high risk of pregnancy and HIV transmission among this population and need for discussion about and provision of family planning services, as well as early discussion of prevention of maternal to child transmission of HIV. Only approximately half of the girls had disclosed their HIV status. Factors that were associated with increased likelihood of pregnancy included: increasing age, married or living with a partner, already a parent at enrolment, and HIV status disclosed at enrolment. The demographics of our cohort overall and those most at risk of pregnancy are consistent with well-established risk factors for adolescent pregnancy, including poverty, child marriage and prior adolescent pregnancy. While beyond the scope of this paper, our data supports global and local calls for widespread policy, community and population health approaches to decrease adolescent pregnancy rates, such as those that target ending child marriage, population-level access to and acceptability of family planning, as well as access to education and sustainable employment for girls and young women [3, 6].

Within this group of HIV positive adolescent girls, a large number (12.9%) were pregnant at enrolment into HIV care, demonstrating that pregnancy itself represents an opportunity to engage adolescents in HIV counseling, testing and treatment. However, this also means that 87.1% were not pregnant and, therefore, represent a group who are engaged in HIV care and could be feasibly targeted for pregnancy prevention as well as other preventative sexual health programming. The overall adolescent pregnancy rate of 55.8 per 1000 women was much higher than that seen in high-income countries, where the pregnancy rate for adolescent girls aged 15–19 between 2010 and 2015 was 22 per 1000, but was lower than the overall adolescent pregnancy rate in Kenya of 96 to 121 per 1000 between 2008 and 2014 [3]. There was a large range of pregnancy rates in adolescents aged less than 15 to age 19, as would be expected, with those in the 18 and 19 year age groups at least double those of younger age groups. Although not a large absolute number, the 15 pregnancies in girls under the age of 15 is important clinically, as these girls are more likely to experience adverse pregnancy outcomes in comparison to older adolescents [9].

To our knowledge, this study represents the largest published analysis of pregnancy incidence among adolescents with HIV in Sub-Saharan Africa. Some of the data used in this analysis was included in a recent IeDEA East Africa analysis of crude cumulative pregnancy incidence 1 year post ART initiation between 2001 and 2009 in women aged 15–50 years; however, our dataset is unique in that it includes younger adolescents, a longer and more recent timeframe, and assesses pregnancy incidence over time in this group along with associated risk factors [22]. Arikawa et al. [23] recently described an incidence rate of 18 per 1000 person years in a cohort of 266 adolescent girls aged 10–19 receiving HIV care in urban Cote D’Ivoire. While this is lower than our incidence rate of 21.9 per 1000 woman years, this may be explained by a lower median age of 12.8 years in their cohort, with 75% of their cohort under the age of 15 [23]. More broadly, in comparison to the literature on pregnancy rates in HIV positive women of all reproductive ages, our overall pregnancy incidence rate of 21.9 per 1000 woman years is lower. Large recent studies in Southern, Eastern and Western Africa have demonstrated pregnancy incidence rates ranging from 29 to 90.7 per 1000 woman years [24–26].

The most interesting and potentially encouraging finding in our study is that pregnancy rates have decreased over the past 10 years in this cohort, both in absolute numbers of pregnancies per 1000 women and in incidence rates that take into account the duration of follow up. Incidence rates are perhaps more informative of the impact of the HIV care program itself as longer engagement in care without an incident pregnancy contributes to an overall decreased incidence rate. This decreasing incidence rate is in contrast to a recent study of HIV positive women in Uganda, aged 15–49, which showed an increase in pregnancy rates from 29.8 to 122 per 1000 woman years between 2006 and 2010 [24]. This study, and others [25–28], have shown increased pregnancy incidence with engagement in care and/or initiation of ART. In women of reproductive age who are not adolescents, this may be a positive trend, demonstrating better control of HIV and ability to conceive. However, in adolescents, regardless of HIV status, the goal is to decrease pregnancy rates and, therefore, we are encouraged by these findings in our program. The data available to us for analysis do not allow us to interrogate factors affecting changing pregnancy rates over time and, therefore, it is not clear whether decreasing pregnancy rates are due to improvement in family planning counselling and uptake or due to other factors, such as worsening health, enrolment of sicker patients or decreasing adolescent pregnancy rates more broadly in the community.

While the current analysis demonstrates a trend in the right direction, the high adolescent pregnancy rates in our cohort compared to high income countries continue to highlight the need for sexual and reproductive health services to be better integrated into the care for adolescent girls with HIV, with a particular focus on access to contraception and adolescent-specific antenatal care programming for those who are first engaged in HIV care during pregnancy. Family planning data in this cohort suggests a high rate of sexual activity, and low rates of use of the most effective forms of contraception (IUD, implants). In addition, family planning method data was only available on 4664/8565 participants and the average age when family planning data was first recorded was 17.2, despite the median age of inclusion in the study of 14.0, both indicating inadequate gathering of sexual and family planning data and, therefore, possible inadequate service provision. Although there were only a small number of pregnancies among those less than 15 years of age, the existence of these pregnancies provides support for discussing family planning with adolescents even at these young ages. Studies in other low-income settings have noted that many adolescents are counselled to simply abstain from sexual intercourse and that health care providers are not adequately trained as to how to provide comprehensive, non-judgmental sexual and reproductive health counselling [7, 29, 30]. As multiple studies have demonstrated and as advocated by the WHO as well as the Kenyan Ministry of Health, HIV positive adolescents are sexually active, are at risk for unintended pregnancy and need integrated family planning and sexual and reproductive health services as an integral component of their HIV care [18, 30–35]. Multi-disciplinary adolescent antenatal and postnatal care has been shown in developed country settings to lead to increased clinic attendance, better birth outcomes (including decreased preterm birth and stillbirth rates), and increased post-partum uptake of contraception [36, 37]. In this population, it may also enable increased engagement in PMTCT and, therefore, decreased rates of vertical transmission [38].

This study has several limitations. First, there is missing data for many of the variables analyzed. Data points related to sex, marriage, and family planning were more likely to be incomplete as these characteristics are only collected on adult data collection forms, whereas the data set was drawn from adult as well as paediatric forms. Missing data may indicate areas where particular groups were not asked (for example, very young adolescents being asked about family planning needs). In addition, because the data are retrospective and gathered from clinical patient files rather than a prospective research study, there is no assurance that data collectors (clinicians) were adequately trained to ask all of the clinical questions and, therefore, there may be inaccurate results. There is also no ability to seek clarification on particular data points with individual patients. Because this study is quantitative, the complex factors that contribute to the recorded outcomes cannot be elucidated accurately. For example, reasons for incident pregnancies, patients’ perceptions of counselling on sexual and reproductive health, stress related to pregnancy, and partner support cannot be assessed. Finally, as this study is only analyzing characteristics of patients within the care program, it is not possible to state whether the results are generalizable to the population of the catchment area as a whole. For example, particular high risk groups (ie. young adolescents) may not seek care due to stigma or lack of agency and therefore, the magnitude of burden of pregnancy in the community may be different than that seen in our study.

## Conclusions

A considerable number of the HIV-positive adolescents in this large HIV care program in Western Kenya presented at enrolment into HIV care as pregnant and many became pregnant as adolescents during follow-up. Pregnancy rates remain high but have decreased from 2005 to 2017. Further research is needed to understand the reasons for these decreasing rates and, in particular, to elucidate clinician and programmatic successes that can be replicated and further developed to continue this trend. Adolescent-focused sexual and reproductive health and ante/postnatal care programs may have the potential to improve maternal and neonatal outcomes as well as further decrease pregnancy rates in this high-risk group. Family planning service integration as well as prospective monitoring and evaluation of uptake are critical next steps to continuing to decrease the adolescent pregnancy rate in this population.

## Data Availability

The datasets generated and analysed during the current study are not publicly available due to lack of Research Ethics Board approval but are available from the corresponding author on reasonable request.

## Abbreviations

AMPATH: Academic Model Providing Access to Healthcare
PMTCT: Prevention of Mother to Child Transmission
ART: Antiretroviral therapy
ASRH: Adolescent Sexual and Reproductive Health
